# Avoiding the pitfalls of gene set enrichment analysis with SetRank

**DOI:** 10.1186/s12859-017-1571-6

**Published:** 2017-03-04

**Authors:** Cedric Simillion, Robin Liechti, Heidi E.L. Lischer, Vassilios Ioannidis, Rémy Bruggmann

**Affiliations:** 1 Interfaculty Bioinformatics Unit and SIB Swiss Institute of Bioinformatics, University of Bern, Baltzerstrasse 6, 3012 Berne, Switzerland; 2 Department of Clinical Research, University of Bern, Murtenstrasse 35, 3008 Berne, Switzerland; 3 Vital-IT, SIB Swiss Institute of Bioinformatics, Quartier Sorge - Batiment Genopode, 1015 Lausanne, Switzerland; 4 SIB Technology, SIB Swiss Institute of Bioinformatics, Quartier Sorge - Batiment Genopode, 1015 Lausanne, Switzerland; 5 Present Address: URPP Evolution in Action; Institute of Evolutionary Biology and Environmental Studies (IEU), University of Zurich, Winterthurerstrasse 190, 8057 Zurich, Switzerland

**Keywords:** GSEA, Gene set enrichment analysis, Pathway analysis, Sample source bias, Functional genomics, Algorithm, R package, Web interface

## Abstract

**Background:**

The purpose of gene set enrichment analysis (GSEA) is to find general trends in the huge lists of genes or proteins generated by many functional genomics techniques and bioinformatics analyses.

**Results:**

Here we present SetRank, an advanced GSEA algorithm which is able to eliminate many false positive hits. The key principle of the algorithm is that it discards gene sets that have initially been flagged as significant, if their significance is only due to the overlap with another gene set. The algorithm is explained in detail and its performance is compared to that of other methods using objective benchmarking criteria. Furthermore, we explore how sample source bias can affect the results of a GSEA analysis.

**Conclusions:**

The benchmarking results show that SetRank is a highly specific tool for GSEA. Furthermore, we show that the reliability of results can be improved by taking sample source bias into account. SetRank is available as an R package and through an online web interface.

## Background

A common feature of many current functional genomics technologies, as well as many different types of bioinformatics analyses, is that they output very large lists of genes, typically in the order of hundreds or thousands. Evidently, interpreting these lists by assessing each gene individually is not practical. Therefore, Gene Set Enrichment Analysis (GSEA) has become the first step in interpreting these long lists of genes. The principle of GSEA is to search for sets of genes that are significantly over-represented in a given list of genes, compared to a background set of genes. These sets of genes consist typically, but not always, of genes that function together in a known biological pathway. In practice, these gene sets are compiled from gene and pathway annotation databases such as GO [1], KEGG [2], Reactome [3], Wikipathways [4], BioCyc [5] or others.

The most naive approach to GSEA is to use a one-sided Fisher’s exact test, also known as hypergeometric test, to determine the significance of over-representation of a gene set in the input list. The drawback of this method is that it requires a clear-cut boundary between included and excluded genes. This distinction may be clear in the case of qualitative experiments such as certain types of proteomics analyses or computational hard clustering analyses. In contrast, other types of analyses, such as most transcriptomics experiments, return a list of *p*-values associated with each gene. These *p*-values express the significance that a gene is differentially expressed between different conditions. Defining a boundary between differentially expressed genes (DEGs) and non-DEGs then relies on applying an arbitrary *p*-value cutoff. Pan et al. [6] have shown that the choice of this cutoff dramatically influences the outcome of a GSEA. As a result, there has been a move away from using hypergeometric methods in favor of other approaches. Tarca et al. [7] have reviewed and benchmarked several of these methods. In their paper, the authors make the distinction between Functional Class Scoring (FCS) methods that calculate a score based on a statistical value, such as *p*-value or rank, for all genes that belong to a given gene set and Single-Sample (SS) methods where for every gene set, a score per sample is calculated. Despite these developments, the hypergeometric method is still widely used, mainly because of its simplicity and because it can be applied to problems other than GSEA. Eden et al. [8] have suggested a method that, rather than applying a global cutoff, determines the optimal cutoff for each gene set. This method was originally used to assess the significance of sequence motifs in promoter sequences [8] but can readily be applied to GSEA as well [9].

As is clear from the previous paragraph, the problem of calculating the significance of a single gene set is well-studied and different adequate solutions exist. However, some other issues still remain unresolved. Arguably, the most important of these issues is that in a typical gene annotation database, many gene sets overlap as a result of genes playing a role in different pathways and processes. Table 1 shows the average fraction of intersecting gene set pairs in several commonly used pathway databases. This table shows that this phenomenon is not only limited to the GO annotation database but occurs in virtually all databases. This seemingly obvious and trivial fact has serious repercussions for GSEA as it confuses the results. Indeed, when several gene sets share a significant proportion of their genes, deciding which gene set from a list of related sets is the most relevant, becomes problematic when no additional information is provided. This problem becomes even more complicated when subset relations exist between the different gene sets in annotation databases. Such relations are most notable with the Gene Ontology [1], but also exist in other databases such as Reactome [3] or KEGG [2]. Several authors have tried to address this problem. PADOG [10] is an FCS-type method that down-weights genes that are part of multiple gene sets. Although the authors show that this approach improves results, from a biological perspective one can argue against penalizing genes simply because they are involved in multiple pathways, since those genes are likely to be key regulators. Other methods have used the explicit graph structure of the Gene Ontology to integrate results. BiNGO [11] simply visualises the GSEA results on top of the Gene Ontology graph. TopGO [12] implements two different ways of using GO structure to improve results. The first method removes genes that belong to a significant set from all its supersets. When a superset is still found to be significant, all of its genes are then also removed from its own supersets and so on. The other method simply down-weights genes belonging to significant subsets. Although these solutions are simple and elegant, they only address subset relations and do not take gene sets into account that only intersect with one another. Jiang and Gentleman (2007) address this problem by calculating for each pair of intersecting gene sets three separate *p*-values: two for the respective set differences and one for the intersection.

**Table 1:** Overview of the extent of overlapping gene sets in some commonly used gene set databases

DB	# sets	median size	% overlap	Min.	p25	Median	p75	Max.
BIOCYC	596	3	3.4%	1.45%	11.61%	20.00%	38.46%	100.00%
GOBP	14524	6	18.2%	0.01%	0.31%	0.76%	1.75%	100.00%
GOCC	1751	7	15.8%	0.01%	0.16%	0.49%	1.45%	100.00%
GOMF	4388	3	6.1%	0.01%	0.17%	0.50%	1.53%	100.00%
KEGG	956	29	13.1%	0.07%	1.61%	4.12%	8.98%	100.00%
Pathway Interaction Database	186	32.5	50.5%	0.52%	1.75%	3.45%	6.38%	63.64%
REACTOME	1784	19	11.8%	0.04%	1.00%	2.44%	6.29%	100.00%
WikiPathways	239	32	26.5%	0.24%	1.31%	2.61%	5.34%	100.00%

Only gene sets with three or more genes were considered. The % overlap column indicates the percentage of gene set pairs sharing at least one gene. The column Min., p25, Median, p75 and Max. list the minimum, 25th percentile, median, 75th percentile and maximum Jaccard values for all pairs of intersecting gene sets

Another problem is that a typical GSEA run returns tens to even hundreds of significant gene sets, depending on the combination of algorithm and databases used. Clearly, when the goal of GSEA is to facilitate the interpretation of long lists of genes, merely converting these into long lists of gene sets helps little to nothing in solving the original problem. An additional difficulty is that correcting for testing multiple gene sets is not feasible using traditional correction methods due to the many overlaps between these sets which violate the independence assumption of these methods. This problem becomes even more complex when one wants to query multiple gene set resources, such as the three different GO domains, biological process, molecular function and cellular component, together with, e.g. Wikipathways and Reactome. One could evaluate a dataset against each of these resources separately but that would only result in disjoint result sets, making the final interpretation even harder. Moreover, combining different gene set resources into a single database and then querying the latter, will result in a very long list of significant gene sets with many overlaps between them as some pathways will be defined in multiple resources. The same principle is also used by Nam et al. [13].

Some methods have been developed to address simultaneously the overlap and the multiple testing problem using generative models [14, 15]. GenGO [14] tries to identify the smallest combination of gene sets that provides the best explanation for the activation of genes in the input list. Although the principle is elegant, this method still requires a cutoff to divide the input gene list into those activated and non-activated genes. Moreover, it also requires an additional arbitrary penalty parameter *a*, which has no empirical meaning. Model-based Gene Set Analysis (MGSA [15]) tries to mitigate these limitations by assuming that the real activation state of a gene is hidden and has to be estimated from the observed data. Although it improves robustness, this change still does not eliminate the cutoff requirement.

Another question is how to determine the background set of genes against which to test for over-representation. Most of the methods discussed above simply use all genes present in the input dataset or even all genes annotated on the genome as the background. Doing so, however, introduces a particular type of bias into the results, which we refer to as sample source bias. Sample source bias occurs when the gene sets returned by GSEA describe the sample rather than the condition being tested. Carefully selecting the background set can eliminate this bias. Although it is arguably an important consideration, surprisingly very few authors have addressed the issue of background selection. To the best of our knowledge, only Maere et al. [11] and Falcon and Gentleman [16] mention this problem.

In this paper, we present SetRank, a novel GSEA algorithm which addresses the overlap and multiple testing problems and allows to query different annotation databases simultaneously. It builds on the idea of Jiang and Gentleman [17] to detect and remove false positive hits based on their overlap with other gene sets. We extend this approach by integrating the remaining results in a *gene set network*. The topology of this network is used to prioritise the final set of results.

Using the same validation method and dataset used by Tarca et al. [7], we show that these different steps dramatically increase the specificity of gene set detection compared to other methods. Apart from this algorithm, we also show how sample source bias can be overcome by defining a correct background set. This step is very straightforward for RNA-seq and shotgun MS-proteomics datasets. For DNA microarray data, it is a little more complicated as this technology does not readily allow to detect presence or absence of a given transcript. Here, we also propose a simple method to define a background set for DNA microarray datasets. We finally demonstrate how defining a proper background set improves the reliability of results.

## Results

### Description of the algorithm

The SetRank algorithm takes as input a gene set collection and a list of genes. The gene set collection can be compiled from different pathway and annotation databases, allowing to query all these resources simultaneously. The list of genes is typically the result of an omics experiment, such as an RNA-seq assay. In most cases, this list will be ranked according to a *p*-value expressing the significance of difference in expression between two conditions. It is also possible to supply an unranked list, e.g. the result of a qualitative proteomics experiment or a genome-wide mutation screen.

In a first step, a primary *p*-value is calculated for every gene set in the collection. Depending on whether the input gene list is ranked according to significance or unranked, a different method is used for this calculation. For ranked gene lists, we use a simplified version of the method described by Eden et al. [8, 9] (see subsection Algorithm details of the Methods section). This method does not depend on an arbitrary cutoff to divide the input gene list into significant and non-significant genes. We choose to not implement the dynamic programming approach proposed by Eden et al. [8] to correct for multiple testing because of performance considerations. In contrast to Eden et al., our algorithm needs to perform many re-calculations of the *p*-value of a single gene set further on (see below).

**Fig. 1:**
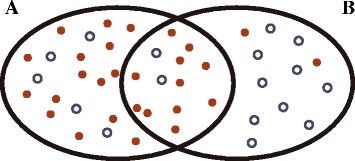
Principle of eliminating false-positive gene set hits. Shown is a Venn diagram of two hypothetical intersecting gene sets. The full dots represent genes determined as significant by a genomics experiment, empty dots represent non-significant genes

After calculating the primary gene set *p*-values, gene sets whose significance is only attributed to the overlap with another gene set, are discarded. This principle is derived from Jiang and Gentleman [17] and is illustrated in Fig. 1. Although both sets shown would initially be considered as significant, it is clear that the significance of gene set B is solely due to its overlap with gene set A. The exact details of this procedure are discussed in Algorithm Details.

This reduction of initially returned gene sets vastly facilitates the interperation of any GSEA analysis. However, there are often still too many different gene sets left after the initial removal step. Moreover, there is often still considerable overlap between any two gene sets, or one set might be a proper subset of another one. These underlying relations make the interpretation of a simple list of gene sets very difficult as the items on the list are not independent of one another. This problem becomes even more apparent when combining different gene set databases.

**Fig. 2:**
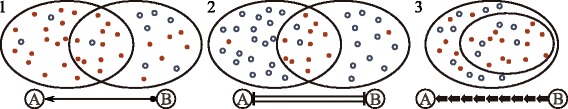
The different edge types in a gene set network (same key as Fig. 1). 1. Normal overlap. Both gene sets are significant but the significance of gene set B is partly due to its overlap with gene set A as the latter has more significant genes. 2. Intersection only. This is the special case where the significance of either gene set is purely due to the intersection between both. 3. Subset. Gene set B is a proper subset of gene set A

**Fig. 3:**
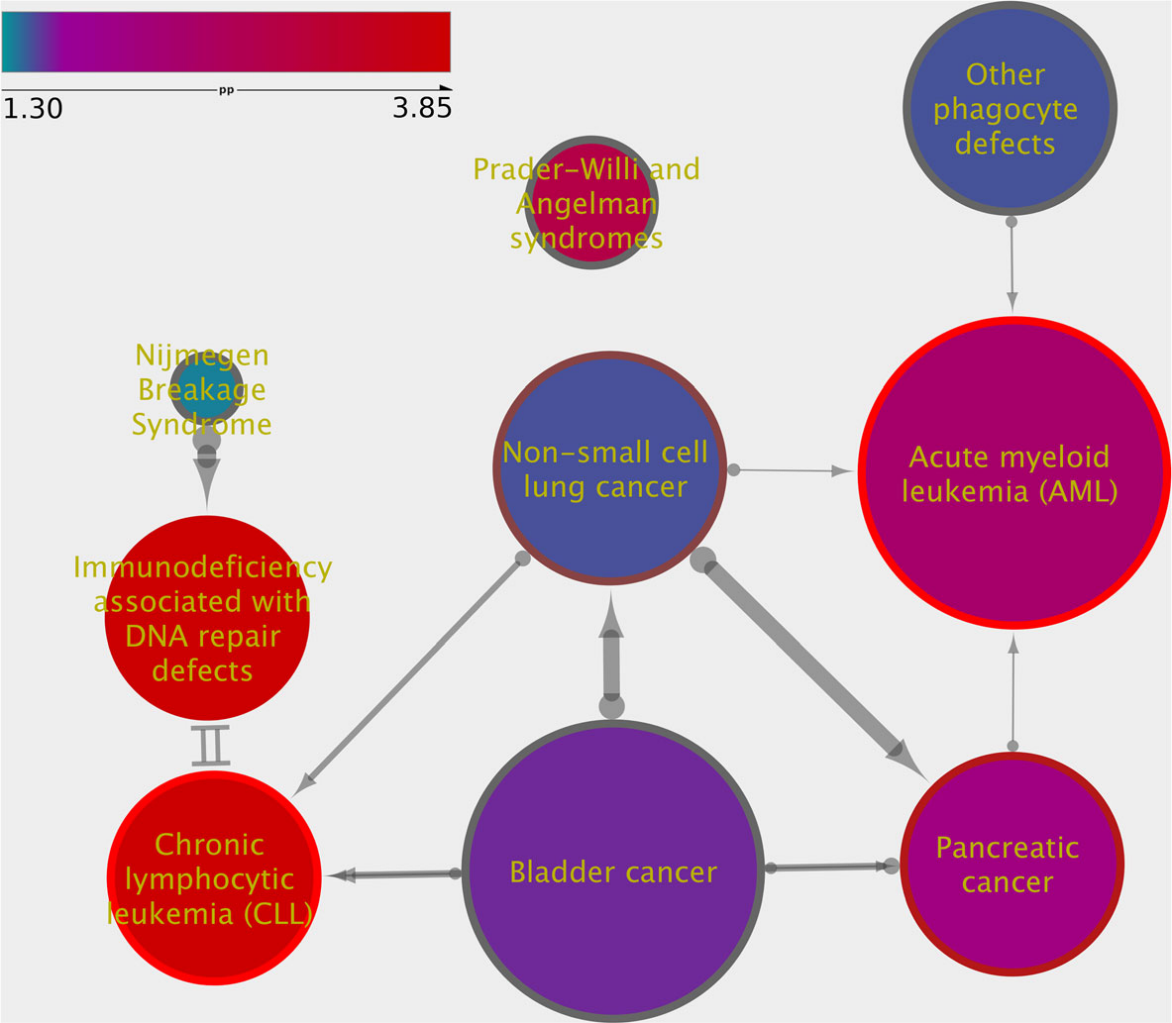
Example of a gene set network returned by SetRank. The node fill color reflects the corrected *p*-value, going from blue to red with decreasing *p*-value (i.e. increasing significance; see inset, pp denotes the negative logarithm of the *p*-value). The node border color reflects the SetRank *p*-value, using the same color coding as the node fill color. Edge thickness reflects the size of the intersection between two gene sets, using the Jaccard similarity. The edge arrows point from the least significant gene set to the more significant one after subtracting the intersection from both of them. A double-line edge indicates a case where the significance of both gene sets was only in the intersection

To facilitate the interpretation of results, we represent the output of the SetRank algorithm as a directed graph which we refer to as a gene set network. In this graph, nodes represent gene sets and edges represent intersections between them. As shown in Fig. 2, we can distinguish between three types of edges. The first and most common one, is an intersection between two gene sets. In this case, it is possible to determine which one of these two sets is the most significant, based on the *p*-value obtained after subtracting the intersection. The resulting edge will then be directed from the less significant to the more significant set. The second type of edge occurs when, after subtracting the intersection from both sets, neither one of them remains significant. This situation arises when the vast majority of significant genes lies in the intersection (see Fig. 2). The third type of edge are cases where one set is a proper subset of another, that is, all elements of the first are also part of the second gene set. Figure 3 shows an example of a gene set network.

Although a gene set network is a very suitable way to visualise the results of SetRank, researchers wishing to understand their dataset can still be left with a very large number of gene sets to evaluate. To help prioritise which gene sets to investigate first, a score, called the *SetRank value*, is calculated for each gene set in a gene set network. This value reflects the prominence of a gene set in a gene set network. It is based on the fact that edges representing normal intersections are drawn from the least to the most significant gene set (see before and Fig. 2). Thus, when a node in a gene set network has a lot of incoming edges, this means that this set is more relevant for a given dataset than a node with a lot of outgoing edges.

The SetRank value is calculated using the PageRank algorithm [18], which is a commonly used method in network analysis. The SetRank value is determined by counting the number and quality of incoming edges to a node to determine the importance of that node. The quality of an edge depends on the SetRank value of the originating node. The SetRank value is thus a recursive score and is determined iteratively. In addition, we also calculate a *p*-value for each node which expresses the signifcance of its SetRank value, given the number of nodes and edges in the gene set network.

### Implementation and availability

**Fig. 4:**
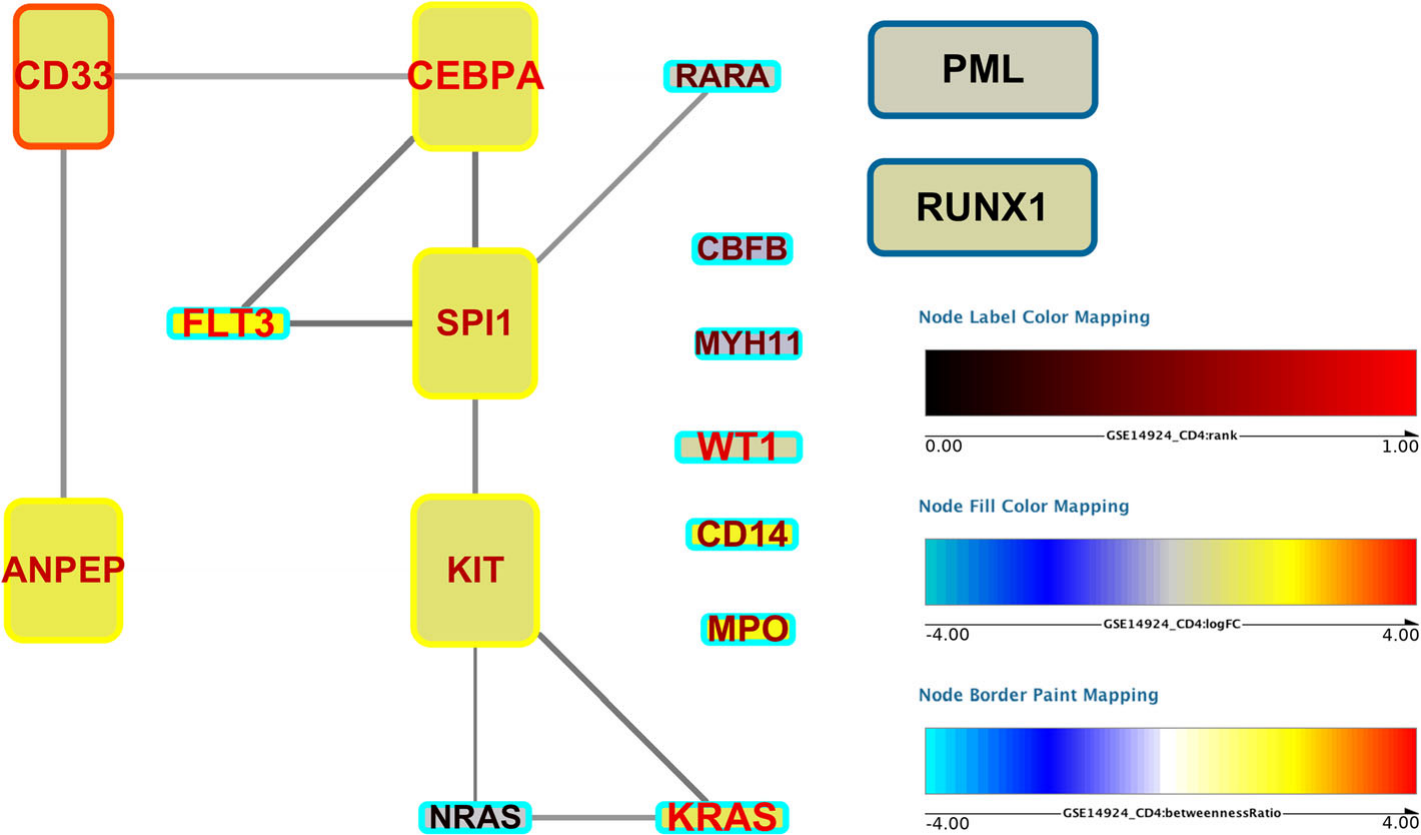
Example of a visualisation of a significant pathway using the SetRank package. Shown are the interactions between genes in the “Acute Myeloid Leukemia (AML)” gene set of the KEGG DISEASE database. The expression data from dataset GSE14924 CD4, which compared AML patients to healthy subjects, is overlayed on top of the network. Node color reflects the log-fold change; the size of the node label reflects the significance of difference in expression; the node label color reflects the gene rank when sorted by *p*-value – a rank of 1.0 means the lowest *p*-value. The node width reflects the global betweenness, i.e. the betweenness of a node in the entire interactome; the node height the local betweenness, i.e. the betweenness in the pathway; the node border color reflects the log-ratio between the local and global betweenness

An implementation of the SetRank algorithm is available as an R package at the CRAN repository. This package provides all functionality to run a SetRank analysis on an input dataset and returns a gene set network. Additionally, it also provides routines to export the results to tabular format and the gene set network as a GraphML file for visualisation with Cytoscape [19]. Apart from visualising the gene set network, the SetRank package also provides functionality to visualise interactions between individual genes inside a significant pathway. To this end, known interactions between genes are first retrieved from the STRING database [20]. The fold-change and *p*-value of each gene in the original input expression data is then visualised on top of the resulting network as node color, ranging from cyan to blue for negative values over black for zero to yellow and red for positive values, and node font size, with bigger fonts for lower *p*-values, respectively. Node size and edge thickness reflect betweenness, which is a measure of network centrality and can help in assessing the importance of a given node in the network. An example of this visualisation is shown in Fig. 4.

Next to this R package, two stand-alone packages are also made available. The first one, GeneSets, can be used to create gene set collections for one or more user-specified organisms. This package consists of a set of scripts that will download data from various online resources and creates for each organism an R package that is ready for use with the SetRank package. The second one, SetRank_interactomes, retrieves species-specific interaction data from the STRING database [20] for use with the gene network visualisation routines. The GeneSets and SetRank_interactomes packages are available from https://github.com/C3c6e6/GeneSets and https://github.com/C3c6e6/SetRank_interactomes respectively.

**Fig. 5:**
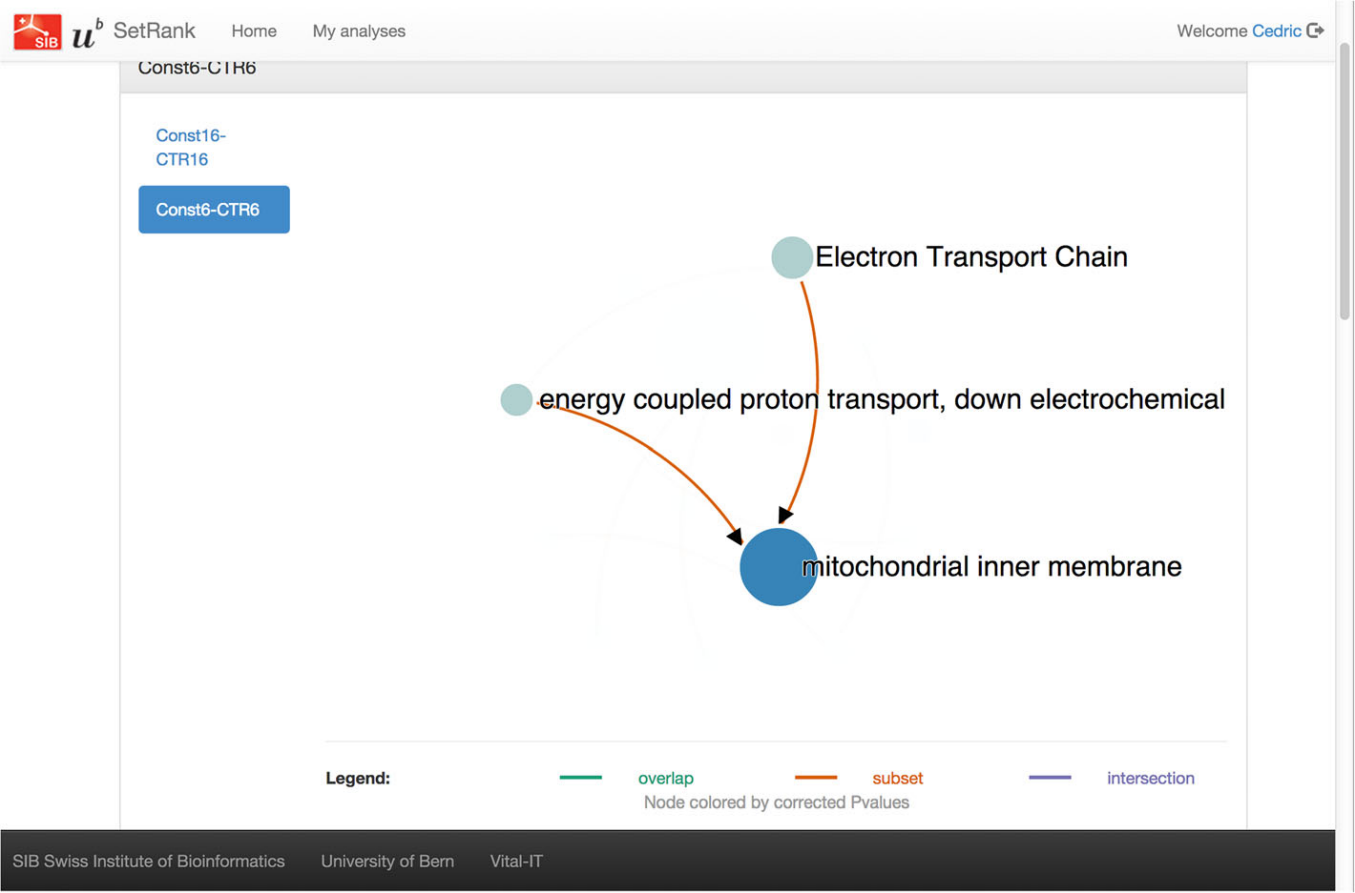
Screenshot of the SetRank web interface

In order to accommodate users that are not familiar with the R programming language, an online interface of SetRank has also been developed and made available at http://setrank.vital-it.ch. This web-interface allows a user to upload one or more tables of gene identifiers with associated *p*-values. Currently the following organisms are supported: *Caenorhabditis elegans*, *Drosophila melanogaster*, *Escherichia coli*, *Homo sapiens*, *Mus musculu*s, *Rattus norvegicus* and *Saccharomyces cerevisiae*. Note that this list may be extended depending on demand from the users. The data is then analysed on the Vital-IT high performance computing infrastructure and the user is notified when the analysis is finished. The resulting gene set networks as well as the gene interaction networks can be visualised within the web browser. Figure 5 shows the visualisation of a gene interaction network of a pathway detected by SetRank using the web interface. All generated results are also available for downloading and off-line analysis with Cytoscape or another graph analysis tool.

### Benchmarking

To assess the performance of the SetRank algorithm we use the benchmarking method proposed by Tarca et al. (2013). These authors have compiled a corpus of 42 microarray datasets that compare patients with a given disease phenotype against healthy individuals. For each of the phenotypes, a matching gene set from the KEGG DISEASE database, referred to as the target set, was identified. Using this information, the performance of a GSEA method can be assessed by how well it identifies the target gene sets in all of the input datasets. Tarca et al. used three different metrics to evaluate performance. The first of these is sensitivity. Tarca et al. used a surrogate score for sensitivity, defined as the median *p*-value of the target set in each dataset.

For simplicity, we will in the remainder of this manuscript refer to this score as sensitivity. Second, prioritisation is defined as the median rank of the target gene set divided by the total number of gene sets tested. Third, specificity, is expressed as the false positive rate at a significance level of 1%. The false positive rate is determined by counting the number of gene sets determined as significant after randomly permutating the genes in all datasets.

We conducted two different SetRank runs on each dataset. In the first run, probesets for which no transcript was detected were filtered out (see below and Methods). In the second run, all probesets were included. These runs are further referred to as the filtered and no-filter runs respectively. The results for each run are shown in Table 2, together with the benchmarks of other methods, taken from Tarca et al.

**Table 2:** Benchmarking results for SetRank compared to other methods

Method	Sensitivity	Prioritization	Specificity
PLAGE [28]	0.0022	25.00%	1.10%
GLOBALTEST [29]	*0.0001*	27.90%	2.00%
PADOG [7]	0.096	*9.70%*	2.50%
ORA	0.0732	18.30%	2.50%
SAFE [30]	0.1065	18.80%	1.30%
SIGPATHWAY Q2 [31]	0.0565	38.00%	0.90%
GSA [32]	0.142	21.00%	1.30%
SSGSEA [33]	0.0808	40.30%	1.00%
ZSCORE [34]	0.095	39.80%	1.00%
GSEA [35]	0.1801	33.10%	2.30%
GSVA [36]	0.1986	51.50%	1.10%
CAMERA [37]	0.3126	43.00%	*0.50%*
MRGSE [38]	0.01	18.80%	4.90%
GSEAP	0.0644	36.20%	15.80%
GAGE [39]	0.0024	35.90%	37.90%
SIGPATHWAY. Q1	0.1165	49.70%	17.20%
SetRank filtered	1.0	*0.75%* (10)	*0.09%*
no filter	1.0	2.05% (10)	–

Scores for other methods are taken from Tarca et al. [7]. The best score in each category is highlighted in italic. Note that the prioritisation score for SetRank is based only on the datasets where the target set could be ranked. The numbers in brackets indicates the number of datasets where this was the case

A problem in calculating these metrics is that SetRank does not rank every gene set from the initial database in the output results as the algorithm discards gene sets at two different checkpoints in the process. As explained in the Methods section, all gene sets with a primary *p*-value above 0.05 are immediately discarded. In addition, gene sets considered as false positives due to their overlap with other sets, are also discarded. In both runs, the target gene sets were ranked in 10 out of 42 datasets. For the sensitivity calculation, we can assign a *p*-value of 1 whenever the target set was not found. As result, the median *p*-value for all runs is 1, simply because the target gene set was not ranked in the majority of datasets. If we, however, define the sensitivity as the median of the initial *p*-values of the target gene sets, we obtain a value of 0.0997, which is largely on par with the other methods.

For the prioritisation, we only calculate the median for those datasets where the target set was ranked. The results show that for the cases where the target set was ranked, the obtained prioritisation score, 0.75% is about one order of magnitude better than the second-best performing method, PADOG, with 9.70%. The false-positive rate of SetRank is 0.09%, which is substantially lower than that of CAMERA, 0.50%. Together, these data show that SetRank has a dramatically improved specificity, both in terms of false-positive rate and of prioritisation, compared to other methods, at the expense of a lower sensitivity.

### Influence of sample source bias on the results

**Fig. 6:**
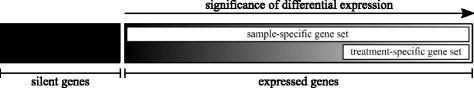
The principle of sample source bias. The black bar represents the group of genes that are not expressed in any sample analysed. The gradient-filled bar represents the set of genes expressed in at least one sample, ordered by significance of differential expression between the different conditions tested. The inset box marked “sample-specific gene set” denotes a gene set that contains genes that are evenly distributed among all significance values of the expressed genes; the one marked “treatment-specific” gene set is highly enriched in significant differentially expressed genes. Note that the box size does not reflect the gene set size but merely the range of significance values of its genes

As mentioned in the introduction, a poorly chosen background set can introduce sample source bias into the dataset. Sample source bias occurs when the gene sets returned by a GSEA algorithm describe the sample rather than the condition. Suppose for instance, that one analyses data from an experiment that compares brain tumour samples against healthy brain samples. Besides general cellular processes such as cell cycle or vesicle transport, a typical gene set database also contains gene sets that describe processes that are tissue or organ-specific, such as synaptic transmission or brain development in the case of brain. Only a few of these processes, if any, will be truly affected by the condition being tested, in this case tumour growth. As explained before, truly affected gene sets are enriched with significant genes, whereas other gene sets contain values drawn uniformly from the entire *p*-value range. The issue of sample source bias arises when tissue-specific gene sets are tested for significance against a background set of genes consisting of the entire genome. Figure 6 illustrates this problem. Indeed, a brain-specific gene set that is not truly affected will still be found to be significant as one compares a list of genes expressed in the brain against those expressed in all other parts of the body. As a result, the returned gene sets will describe both the condition being tested as well as the brain itself. If, on the other hand, one uses a background set containing only genes that are expressed in the brain, the returned gene sets will be specific only to the condition tested.

In the ideal case, the perfect background set consists of all genes that can be expressed in the tissue type under study. Several resources have already been developed to characterise the complete expression profiles of several human [21] or animal [22] tissues under normal conditions. Although these are useful resources, they fall short when one wants to compare abnormal tissue, such as tumor samples, or very specific cell types such as immune cell populations. The next best solution to construct a background set is to take all genes that are positively identified at least once in any of the sample groups in the dataset being analyzed. When using a technology that detects absolute transcript or peptide levels, such as RNA-seq or shotgun proteomics, a background set is easily compiled. In the case of DNA microarray datasets, the solution is less straightforward as there is no definite cutoff value for the expression intensity of a probe set to determine if a transcript is detected or not. One can, however, expect that the intensities of a probe set for which a transcript is not present in any of the samples in the dataset, will have both low mean and variance values. Using this principle, we devised a simple scheme to remove undetected transcripts from the dataset (see Methods).

The influence of background selection is not obvious when only evaluating gene sets from the KEGG DISEASE database. Indeed, although there is a difference between the median prioritisation scores (see Table 2), there is no overall significant difference between both the *p*-values (Mann–Whitney test, *p* = 0.15) and the prioritisation scores (*p* = 0.55) of the filtered and unfiltered runs. This lack of effect is probably due to the fact that the KEGG DISEASE gene sets only describe specific diseases and not tissue-specific processes.

To demonstrate the effect of sample source bias properly, we re-analysed the benchmarking datasets with a more general purpose gene set database, the Biological Process terms of the Gene Ontology (GOBP). For this analysis, we only considered those datasets that are derived from brain tissue, as this is the most abundant tissue type in the entire corpus of benchmarking datasets (9 out of 42). Each dataset was analysed with SetRank using three different background sets. The first background set consisted of all genes represented on the microarray used, the second had all genes considered to be not expressed removed, and the third had an equal amount of randomly selected genes removed. The Setrank runs conducted with these background sets are respectively referred to as the ‘no filter’, ‘filter’ and ‘random filter’ runs.

The aim of this analysis is to investigate to which extent the choice of background set influences the occurrence of brain-related terms in the output of SetRank. We hypothesise that using a tissue-specific background set will reduce the occurrence of brain-related gene sets in the output of SetRank. To test this hypothesis, we compared the significance of brain-specific GO terms between these three runs. Here, a brain-specific term is defined as any GO term that inherits from the ancestral terms “nervous system development” (GO:0007399), “neurological system process” (GO:0050877), or “synaptic transmission” (GO:0007268). Note that there are other terms, such as “regulation of neuron apoptotic process” (GO:0043523) that are clearly related to brain function but inherit from more general process terms such as “regulation of apoptotic process” (GO:0042981) and are scattered all over the GO graph. These terms are not considered here as they can not be readily included using objective criteria.

The results of these three runs for one dataset (GSE19728), which compares brain tumour samples to healthy brain tissue, are shown in Table 3. Both the ‘no filter’ and ‘random filter’ runs have four brain-specific terms among the ten top ranked gene sets, whereas the ‘filter’ run only has one. The results for this single dataset already indicate that the choice of background set plays a role in reducing sample source bias as the four brain-specific terms in the ‘no filter’ and ‘random filter’ terms most likely only describe the sample, i.e. the brain, rather than the condition being tested.

**Table 3:** The effect of sample source bias on dataset GSE19728

	No filter	Filter	Random filter
1	*regulation of synaptic transmission* (GO:0050804)	*neurotransmitter transport* (GO:0006836)	*regulation of synaptic plasticity* (GO:0048167)
2	*neuron-neuron synaptic transmission* (GO:0007270)	regulation of exocytosis (GO:0017157)	*pallium development* (GO:0021543)
3	regulation of Rho protein signal transduction (GO:0035023)	regulation of transporter activity (GO:0032409)	*telencephalon development* (GO:0021537)
4	establishment of vesicle localization (GO:0051650)	membrane depolarization (GO:0051899)	positive regulation of cell development (GO:0010720)
5	*synaptic vesicle transport* (GO:0048489)	regulation of ion transmembrane transporter activity (GO:0032412)	membrane depolarization (GO:0051899)
6	positive regulation of cell development (GO:0010720)	positive regulation of cell development (GO:0010720)	establishment of organelle localization (GO:0051656)
7	*regulation of postsynaptic membrane potential* (GO:0060078)	establishment of vesicle localization (GO:0051650)	regulation of Rho GTPase activity (GO:0032319)
8	regulation of Rho GTPase activity (GO:0032319)	positive regulation of cell projection organization (GO:0031346)	*synapse organization* (GO:0050808)
9	regulation of neuron apoptotic process (GO:0043523)	peptidyl-serine phosphorylation (GO:0018105)	vesicle localization (GO:0051648)
10	membrane depolarization (GO:0051899)	regulation of peptide secretion (GO:0002791)	establishment of vesicle localization (GO:0051650)

Brain-specific GO terms are marked in italic.

**Fig. 7:**
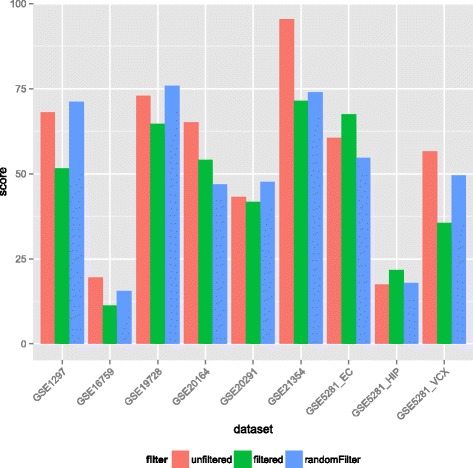
The influence of background selection on brain-tissue derived datasets. The height of the bars indicates the score which represents the total significance of all brain-related gene sets. See the main text for details

To investigate if this trend is persistent across all nine brain datasets, we calculated for each SetRank analysis the negative logarithm of the product of the corrected *p*-values of all brain-specific GO terms detected as a score for the total significance of these terms. These scores are shown in Fig. **7**. Comparing these scores with a one-tailed Wilcoxon signed rank test, we see that scores of the ‘filter’ runs are significantly lower than those of the ‘no filter’ runs (*p* = 0.02), whereas there is no significant difference between the ‘no filter’ and ‘random filter’ runs (*p = 0.10*). These results demonstrate that removing non-expressed genes from the background set helps to eliminate sample-specific gene sets from the analysis results and that this elimination is not due to a loss in statistical power caused by a smaller background set.

## Discussion

Assessing the performance of a GSEA method is not straightforward as it is hard to know beforehand which gene sets should be considered as true positives. Many authors have used simulated data to validate their method [14, 15, 23]. The validity of this approach is however debatable, as one can tweak the simulation model to give optimal results. The strategy proposed by Tarca et al. [7] which we used here, provides an objective benchmark to evaluate different methods. It should be stressed though that this approach is by no means perfect. Indeed, the KEGG disease database consists of collections of genes implicated in specific diseases, including many genomic biomarkers. As the benchmarking approach suggested by Tarca et al. is based on analysing transcriptomics data, one can only detect the target gene set when the expression levels of the included biomarkers are sufficiently affected, which is an unlikely assumption. Therefore, there is no guarantee that the designated target gene set in the benchmarking corpus for a specific disease is actually the most relevant one. Nevertheless, this approach allows at least to assess the relative performance between different methods.

As mentioned in the description of the algorithm, we have chosen not to implement the multiple testing correction method used by Eden et al. [8] when calculating the initial *p*-value of a gene set. Instead, our method detects false-positive hits by discarding gene sets based on their overlap with other sets. The very low false-positive rate observed in the benchmarking results clearly shows that this approach is very effective. Thus, our approach of false-positive detection can be seen as a data-driven form of multiple-testing correction.

As can be seen from the benchmarking statistics, the sensitivity, as expressed in median *p*-value of the target sets, of our method is rather poor at first sight, with a value of 1. As mentioned above, this score is mainly an artifact of our algorithm not assigning a *p*-value to all input gene sets. If we look at the median of the initial *p*-values of the target sets, we obtain a value of 0.0997, which is still better than 6 out of 16 of the methods listed in Table 2.

In practice, the limited sensitivity of SetRank is unlikely to be a problem as the number of gene sets initially returned by GSEA typically runs in the hundreds, especially when querying multiple databases. Indeed, during our analysis of the brain-specific datasets using only the GOBP database, we found on average 333 initially significant gene sets, with a standard deviation of 34.7. Moreover, the gene sets in the KEGG DISEASE database are a lot smaller (average size 4.68) compared to other databases such as GOBP (average size 80.0) which further diminishes the issue of limited sensitivity as larger gene sets are more readily detected due to increased statistical power. Note, however, that SetRank is still capable of detecting small gene sets. Indeed, in our analysis of the brain-specific datasets with the’filter’ runs, we detected on average 131 significant gene sets with 10 or less genes (standard deviation 10.5) and 76 gene sets with 3 or less genes (standard deviation 7.7). Finally, it should be noted that for 12 out of the 16 methods benchmarked by Tarca et al., the median *p*-value for the target gene set is higher than 0.05. This observation means that, for the majority of datasets in the benchmarking corpus, these methods would not detect the target sets either when applying a *p*-value cutoff of 0.05 on the returned gene sets. Thus, in practice, the sensitivity of our method is on par with the bulk of the other GSEA methods available.

Contrary to the sensitivity, the statistics for prioritisation and false-positive rate show that our method significantly outperforms all other methods available. A prioritisation score of 0.75% on KEGG DISEASE, a gene set database with 522 gene sets, means that our method ranks the target gene set consistently within the top 4 ranked gene sets, whenever it is detected. As this result is only based on 10 out of 42 benchmarking datasets, a direct comparison of this score with the other methods is unfair. Nevertheless, taken together with the very good specificity score, the low prioritisation score does demonstrate that, whenever SetRank does assign a low rank to a gene set, it is very likely to be relevant. One reason for this excellent performance is that we do not order the final results on corrected gene set *p*-value alone but also on the topology of the gene set network. The advantage of this approach is illustrated in Fig. 3 which visualizes the results on dataset GSE14924_CD4, which compares the transcriptomes of patients with Acute Myeloid Leukemia to those of healthy subjects. Although the target gene set “Acute Myeloid Leukemia” does not have the lowest *p*-value, as can be seen from the node fill color, it has the highest SetRank value and is consequently ranked first in the final result list.

The obtained specificity value, 0.09%, is again much lower compared to all other methods and is probably due to the FDR correction applied by our algorithm on the different connected components in the gene set network. Other GSEA methods do not perform FDR correction, as the latter assumes statistical independence of the entities tested, which is clearly not the case for intersecting gene sets. However, this assumption does hold in the case of separate network components as they, by definition, do not share any genes.

The SetRank algorithm itself is controlled by two parameters. The first one is the *p*-value cutoff applied to discard non-significant gene sets in the first filter step as well as during the elimination of false positives. During our benchmarking analysis, we have set this value to 0.05. Although setting this parameter is an arbitrary decision, its value has little effect on the overall performance of SetRank. Indeed, applying a stricter cutoff, only affects the sensitivity performance of our method, as fewer gene sets will be finally ranked. As discussed before, the prioritisation score mainly depends on the topology of the gene set network. Although a stricter cutoff results in fewer nodes, the edge directions, which are used to determine the SetRank score of a node, of the remainig nodes are not affected. The second parameter, the FDR cutoff, has no effect on neither the sensitivity nor the prioritisation. The specificity in our benchmarking is defined here as the number of false positive results at a significance level of 1%. As a result, the FDR cutoff also has no influence on this metric. Apart from these, the process that builds the gene set collection has one additional parameter, the maximum size of gene sets to include. This parameter was added to avoid the inclusion of vague, general gene sets, such as “metabolic process” into the results, at the expense of more precise, smaller sets.

The analysis of the influence of sample source bias illustrates the importance of defining an appropriate background set. To the best of our knowledge, this paper is the first to include a thorough analysis of this phenomenon. Our analysis shows that removing non-expressed genes from the background set significantly reduces the occurrence of tissue-specific gene sets in the output of GSEA. Note that we do not want to exclude these gene sets *a priori*, but to ensure that, when they turn up as significant, their significance is actually due to the effect being measured. Thus, the few tissue-specific gene sets that remain after applying a corrected background set can then be interpreted as processes that are genuinely affected by the condition studied. The benchmarking results for the filter run show no significant loss of statistical power resulting from smaller background sets, as the target set is detected in just as many cases.

## Conclusion

The combination of excellent prioritisation and specificity scores, ensures that SetRank returns either reliable results or no results at all, making it a highly reliable method for GSEA and ideally suited for querying multiple gene set databases simultaneously. This reliability can be further improved by eliminating sample source bias.

## Methods

### Algorithm details

#### Calculating primary gene set *p*-values

Depending on whether the input gene list is ranked or not, a different method is used to calculate the primary *p*-values of all gene sets in the input gene set collection. For ranked gene lists, we use a simplified version of the method described by Eden et al. [8, 9] This method takes as input the list *L* of all genes present in the dataset, ranked by increasing *p*-value and a gene set *S* ⊂ *L*, also ranked in the same way as *L*. Let *l* and *s* denote the sizes of *L* and *S* respectively. Further, let, for a gene *g*_i_ Є *S*, *i* Є {1, …,*s*} denote the rank of *g*_*t*_ in *S* and *l*_*t*_ the number of genes in *L* that rank above or equal to – i.e. have a *p*-value less or equal to – *g*_*t*_. The *p*-value of gene set *S* is then given by

$$ p= \min {P}_F\left(\begin{array}{cc}\hfill i\hfill & \hfill s- i\hfill \\ {}\hfill {l}_i- i\hfill & \hfill {l}_i-\left({l}_i+ s- i\right)\hfill \end{array}\right) $$

where *P*_*F*_ is the *p*-value obtained by applying a one-tailed Fisher exact test on the 2 × 2 contingency matrix. The lowest *p*-value obtained for all values of *i* is then used as the final *p*-value.

The original method by Eden et al. [8] uses a sophisticated approach to correct for multiple testing by applying a dynamic programming algorithm. For reasons of computational efficiency, we do not correct for multiple testing at this stage.

For methods that do not provide a means of ranking genes according to significance scores, such as clustering analysis, a Fisher exact test can be used to calculate the *p*-value of a gene set. In this case, the input consists of the same list *L* and gene set *S* as used above as well as an additional cluster of genes C ⊂ *L*. Let *l*, *s*, and *c* denote the sizes of *L*, *S*, and *C* respectively and let *n* = |*C* ∩ *S*|. In this case, the *p*-value that expresses how significantly *S* is over-represented in *C* compared to all of *L*, is given by

$$ p={P}_F\left(\begin{array}{cc}\hfill n\hfill & \hfill s- n\hfill \\ {}\hfill c- n\hfill & \hfill l-\left( c+ s- n\right)\hfill \end{array}\right) $$

After calculating the primary *p*-values, we discard all gene sets with a *p*-value above a user-defined threshold. To avoid false negatives – i.e. gene sets that are wrongfully discarded – we recommend to only apply a loose cutoff in this phase, typically in the range of 0.01 – 0.05.

#### Eliminating false positives

The procedure to eliminate false positive gene sets involves testing all pairwise combinations of the initially retained gene sets. As a result, the computation time scales quadratically with the number of retained gene sets. To reduce calculation times, we first compute the list of set pairs whose intersection is significantly larger than expected by chance so as to only compare pairs of gene sets that are biologically related. This filtering step is accomplished by calculating for each pair of sets *M* and *N* with sizes *m* and *n*, both subsets of a larger set *L* with size *l*, the probability *p* that they share at least *i* elements by chance, as given by:

$$ p={P}_F\left(\begin{array}{cc}\hfill i\hfill & \hfill n- i\hfill \\ {}\hfill m\hfill & \hfill l-\left( m+ n- i\right)\hfill \end{array}\right) $$

Only pairs with a *p*-value below a certain cutoff are retained. Again, we only use a permissive cutoff value here, typically 0.01, without any correction for multiple testing.

In the next step, we iterate over the obtained list of significant set pairs to detect false positive gene sets. For every pair of sets *A* and *B*, we recalculate for each set a new *p*-value, *p*′, this time using only the set differences *A*′ = *A*\*B* and *B*′ = *B*\*A*. If the value *p*′ of gene set *A* is higher than the previously used cutoff and the *p*′ of *B* stays below this cutoff, then this means that the significance of *A* was purely due to its overlap with *B* (see Fig. 1). Gene set *A* is not immediately discarded but the fact that it was invalidated by gene set *B* is recorded. When *A* is a proper subset of *B*, only the *p*-value of *B* will be re-evaluated.

After evaluating all gene set pairs in this manner, the obtained information is used to construct a directed graph where every node represents a gene set and edges denote the previously determined invalidating relations with the edges pointing to the invalidated nodes. The purpose of this graph is to determine which gene sets can be discarded as false positives. In the case of *A* → *B*, where *A* has no incoming edges and *B* none outgoing, *B* will be discarded. In the more complicated case of *A* → *B* → *C* →, where A has no incoming edges, only *B* will discarded and *C* will be retained. This process is repeated until no more gene sets can be discarded. Note that in the special case of *A*↔*B* neither set is discarded as in this situation the majority of significant genes lies in the intersection of both sets. Such cases are flagged and indicated in the program output (see next section).

### Gene Set network and prioritisation of gene sets

The results of the algorithm are stored in a gene set network which can be visualised and analysed using network analysis software tools such as Cytoscape [19]. Note that this network is different from the graph created in the previous section which only contained invalidating relations. In the gene set network, all remaining gene sets are represented as nodes. Edges are drawn between any two nodes of intersecting gene sets. We distinguish between three different types of edges: subsets, intersections, and overlaps (see Fig. 2). A subset edge is created when one gene set is a proper subset of the other and is directed from the subset to the superset. Intersection edges represent the special cases *A*↔*B* from the previous section where the genes that make both gene sets significant are only located in the intersection. This type of edge has no direction. Overlap edges denote all other cases of intersecting gene sets. The direction of an overlap edge is towards the node with the strongest, i.e. lowest, associated *p*′ value as discussed in the previous section.

To prioritise the collection of remaining gene sets, a few additional values are calculated for each node. The first two are the SetRank value and the associated *p*-value. The SetRank value is determined by calculating the PageRank value for each node, considering only the overlap edges in the gene set network and using a damping factor of 0.85. Next, the PageRank values from 100 randomly generated networks with the same node and edge count as the original network, are determined. These values are then fit to a normal distribution to determine the SetRank *p*-values for all nodes in the original gene set network, which are subsequently adjusted for multiple testing using the Holm [24] method.

The corrected *p*-value of a gene set *A* is determined by calculating the value *p*′ (see above) for each overlapping gene set *B*, i.e. each neighbor node in the gene set network, and taking the maximum of these values. Finally, a multiple testing correction is applied on each of the weakly connected components. First the *p*-value of a component is determined by taking the minimum corrected *p*-value of all its nodes. Second, the Holm method is used again to correct the component-level *p*-values for multiple testing.

The final results are first ordered by SetRank *p*-value, then by adjusted *p*-value and finally by corrected *p*-value.

### Benchmark analysis

All hybridisation CEL files for all 42 microarray datasets used by Tarca et al. [7] were downloaded from the Gene Expression Omnibus [25] website. All arrays were annotated with either the HGU133A_Hs_ENTREZG or the HGU133Plus2_Hs_ENTREZG chip definitions provided by Dai et al. [26] and subsequently background-corrected and normalised using the RMA method [27].

The KEGG disease gene set definitions were downloaded using the KEGGRest Bioconductor package. Tarca et al. [7] have identified for each of these datasets a target KEGG disease gene set.

SetRank was run on each dataset using the default settings, meaning in ranked mode, with an individual set *p*-value cutoff of 0.05, and an FDR cutoff of 0.05 using the KEGG DISEASE gene sets. Two different runs of SetRank were conducted on each dataset. In the first run, referred to as the ‘filter’ run, probesets for which no transcript was detected were filtered out (see below). In the second run, referred to as the’no filter’ run, all probe sets were included.

### Detection calling on microarray datasets

To detect if a transcript for a given gene is detected in any of the samples in a microarray dataset, we used a simple filtering approach based on two cutoffs. The first cutoff is the median of all relative median absolute deviations (rMAD) for all probesets in a dataset. The rMAD is defined as the median absolute deviation divided by the mean. Note that this value is calculated on the non log-transformed expression intensities. The second cutoff is the 5th percentile of the mean expression value, calculated on the log-transformed expression intensities, of all probesets with an rMAD higher than the median. All probesets with rMADs and means below both cutoffs are then considered to have not been detected in any of the samples.

### Sample source bias analysis

For the sample source bias analysis, the 9 datasets from the benchmarking corpus that are derived from brain tissue were again analysed with SetRank, this time using gene sets from the Biological Process domain of the Gene Ontology [1], using the same settings as before. This time, three runs were conducted on every dataset. Two of these were a ‘filter’ and ‘no filter’ run, as described above. The third run was a ‘random filter’ run where the same number of probesets was discarded as in the ‘filter run’, only this time the probesets were randomly chosen.

## Abbreviations

DEG: Differentially expressed genes
FCS: Functional class scoring
GOBP: Gene ontology biologial process
GSEA: Gene set enrichment analysis
MGSA: Model-base gene set analysis
rMAD: Relative median absolute deviation
SS: Single sample method
